# Meta-research matters: Meta-spin cycles, the blindness of bias, and rebuilding trust

**DOI:** 10.1371/journal.pbio.2005972

**Published:** 2018-04-02

**Authors:** Lisa Bero

**Affiliations:** Charles Perkins Centre, Faculty of Pharmacy, The University of Sydney, Sydney, Australia

## Abstract

Meta-research is research about research. Meta-research may not be as click-worthy as a meta-pug—a pug dog dressed up in a pug costume—but it is crucial to understanding research. A particularly valuable contribution of meta-research is to identify biases in a body of evidence. Bias can occur in the design, conduct, or publication of research and is a systematic deviation from the truth in results or inferences. The findings of meta-research can tell us which evidence to trust and what must be done to improve future research. We should be using meta-research to provide the evidence base for implementing systemic changes to improve research, not for discrediting it.

‘That’s so meta!’ exclaimed my student. And I thought, ‘Now she’s really got it!’ We were working on a review of studies of ‘spin’—research reporting practices that distort the interpretation of results and mislead readers by making findings appear more favourable than they are. At the time of our epiphany, we were discussing how to avoid putting spin on the conclusions of our review of studies of spin [1]. If that’s not ‘meta’, I don’t know what is [2]. Meta-research may not be as click-worthy as a meta-pug—a pug dog dressed up in a pug costume—but it is crucial to understanding research.

Meta-research is research about research. A particularly valuable contribution of this discipline is identifying biases in a body of evidence. Bias can occur in the design, conduct, or publication of research and is a systematic deviation from the truth in results or inferences. The findings of meta-research can tell us which evidence to trust and what must be done to improve future research.

While biases related to internal validity, such as appropriateness of randomisation or blinding, can be detected in a single study, the impact of such biases on a research area can only be determined by examining multiple studies. By examining the association of the appropriateness of randomisation in clinical trials with the magnitude of the intervention effect estimate in multiple groups of studies across different clinical areas, for example, it’s been shown that inadequate sequence generation or concealment of allocation will overestimate effect estimates by about 7% and 10%, respectively [3]. This means that when we come across the findings of a randomised controlled trial on any topic and see that the randomisation is inadequate, we have empirical evidence to support scepticism about the findings. In such cases, the results are probably exaggerated.

Meta-research has also quantified the biases associated with inappropriate randomisation and blinding in trials with animals (see Fig 1). Analyses of preclinical animal studies examining interventions for stroke, multiple sclerosis, and trauma have shown that lack of randomisation and blinding, inadequate statistical power, and use of comorbid animals are associated with inflated effect estimates of the tested interventions [4, 5].

**Fig 1:**
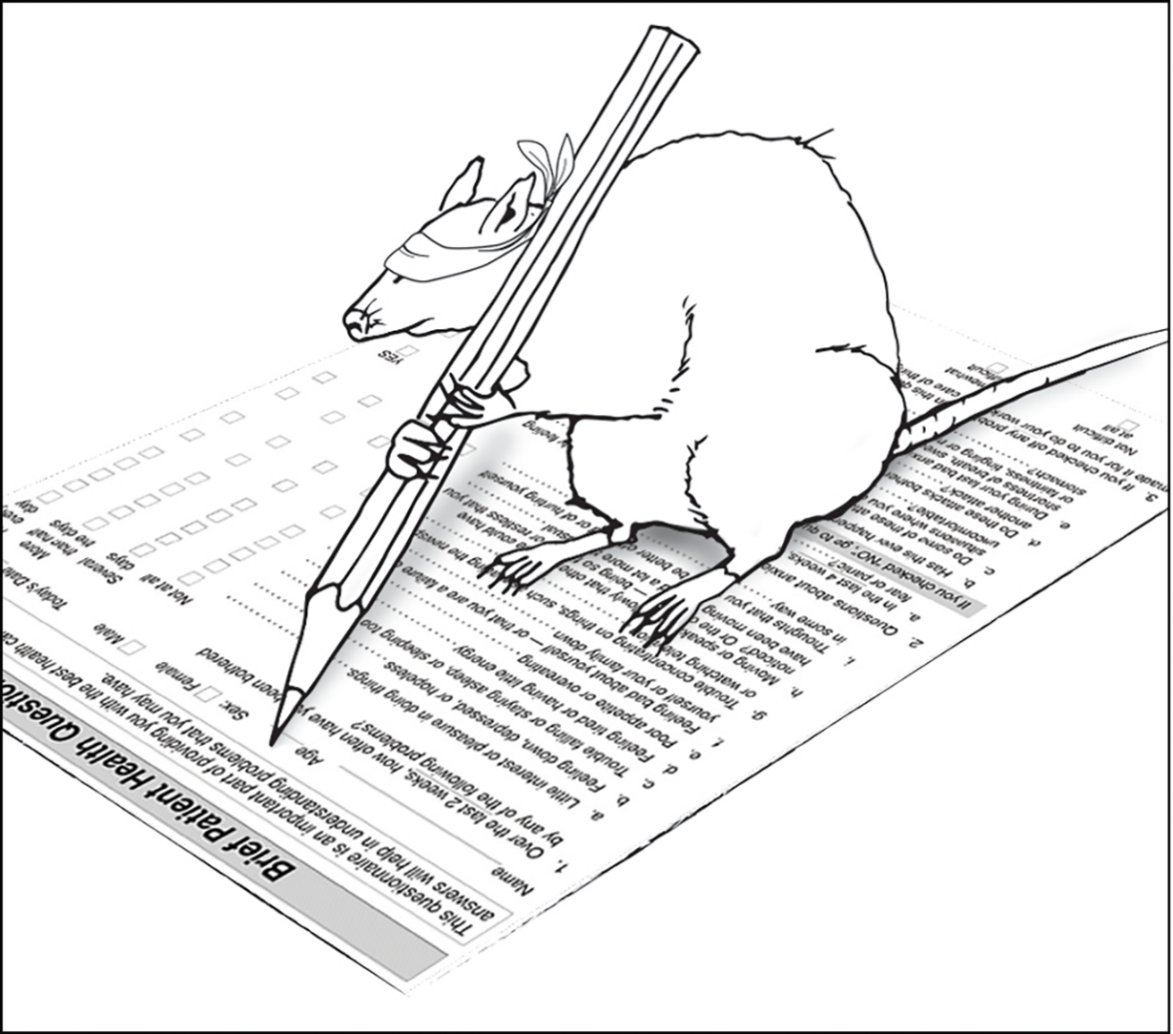
To avoid bias, the mouse was blinded when self-reporting outcomes. Image credit: Lorris Williams.

Meta-research has been used to identify important biases in research that are not related to study methodology, such as publication bias, outcome reporting bias, funding bias, and spin. Reporting bias, including the failure to publish entire studies or the selective reporting of outcomes or analyses in published studies, has been detected across a variety of health fields and among animal studies [6–8]. This results in the published body of evidence being overwhelmingly ‘positive’ or showing statistically significant results. Researchers, not editors, are usually to blame, as publication bias often results from a failure to submit negative studies, not from journals rejecting them [9, 10].

Funding bias has been demonstrated in pharmaceutical, tobacco, chemical, and other research areas. For example, a Cochrane meta-research review included 75 studies examining the association between sponsorship and research outcomes of drug or device studies across different clinical areas. Industry-sponsored studies more often had efficacy results, relative risk (RR): 1.27 (95% CI 1.17–1.37); harm results, RR: 1.37 (95% CI 0.64–2.93); and conclusions, RR: 1.34 (95% CI 1.19–1.51) that favoured the sponsor [11]. Industry- and non-industry-sponsored studies did not differ in methodological biases, such as random sequence generation or follow-up, although industry-sponsored studies were at a lower risk of bias for blinding. This suggests that other factors, such as the use of unfair comparator doses or the failure to publish studies with unfavourable results, are associated with the different outcomes of industry- and non-industry-sponsored studies. Existing tools for assessing risk of bias or internal validity of studies are not sufficient for identifying the mechanisms of bias associated with funding source.

Meta-research has been critical in identifying biases in interpretation, known as ‘spin’. Our review found 35 reports that had investigated spin in clinical trials, observational studies, diagnostic accuracy tests, systematic reviews, and meta-analyses [1]. The highest prevalence of spin was in trials. Some of the common practices used to spin results included detracting from statistically nonsignificant results and inappropriately using causal language. Current peer review practices are not preventing spin, so editors and peer reviewers should become more familiar with the prevalence and manifestations of spin in their area of research.

Has all of this navel-gazing by researchers been useful? Meta-researchers interested in bias may have pulled the rug out from under themselves. Because meta-research is such a great tool for detecting biases that are present across a body of evidence, it has led to proclamations that all research is biased and should not be trusted. Rather than using the findings of meta-research on bias to discredit research, we should use them to identify what needs to be fixed. We have made great strides towards systemic solutions, particularly in the area of clinical research. The identification of biases associated with industry conflicts of interest has led to policies for disclosure, management, and elimination of the conflicts. Compliance with mechanisms to reduce publication and selective reporting bias—such as study registration, protocol availability, and open access to data—is now required by many clinical journals as a condition of publication. Tools for assessing risks of bias in studies have been informed by empirical investigation of methodological biases and are being improved based on empirical evidence. As meta-research discovers biases in a variety of fields, we need to expand and adapt the solutions we are using in clinical research to reduce bias in all areas.

Ironically, the identification of biases in bodies of evidence has led to criticisms of the use of another type of evidence synthesis—systematic review and meta-analysis—to support policy decisions and guidelines. The argument is that if all research is flawed, why should we bother to summarise it? In the not-too-distant past, if you were seeking advice on a medical condition from a healthcare practitioner, they would likely have been informed by practice guidelines developed using the ‘good old boys sit around the table’ (GOBSAT) method [12]. It was the opinions of healthcare practitioners, often supported by their own financial conflicts of interest, that formulated the recommendations made in clinical practice guidelines [13]. Nowadays, healthcare practitioners are more likely to be informed by guidelines based on multiple systematic reviews and meta-analyses addressing all the questions relevant to the guideline [14]. High-quality reviews always include an assessment of the risk of bias of the included studies so that it is transparent that recommendations are based on strong, weak, or no evidence [15]. Thus, meta-research has boosted the quality of systematic reviews by enabling the identification of biases in the included studies.

Meta-research will point out the imperfections in a body of evidence. It will identify the flaws so we can focus on the best available evidence. Methodological advances in how we do systematic reviews and meta-analysis will provide better ways to deal with the biases identified. Now that we know the extent of publication bias across various fields, we should not limit meta-analysis to only published, peer-reviewed data. For years, Cochrane has been recommending searching for unpublished data as part of a comprehensive search strategy [15]. Meta-research has provided more specific guidance by showing that certain types of meta-analyses, such as those of drug efficacy or harm, should include data from clinical study reports and regulatory databases [16, 17].

Identifying biases in research will help us identify systemic changes that are needed to improve the quality and trustworthiness of research. We should be using meta-research to provide the evidence base for implementing these changes, not for discrediting research. When using meta-research to bolster criticisms of systematic reviews or meta-analysis for informing health decisions, we need to think carefully about the alternatives. I’m not ready to turn the clock back to making decisions based on the GOBSAT method. Give me the best available evidence any day.

## Abbreviations

GOBSAT: good old boys sit around the table
RR: relative risk
